# 2776. Decrease in ceftriaxone treatment failure rates for infections caused by inducible AmpC producing *Enterobacterales* associated with changes in laboratory susceptibility testing reporting

**DOI:** 10.1093/ofid/ofad500.2387

**Published:** 2023-11-27

**Authors:** Ayesha Khan, Romney Humphries

**Affiliations:** Clínica Alemana de Santiago, Santiago, Region Metropolitana, Chile; Vanderbilt University Medical Center, Nashville, Tennessee

## Abstract

**Background:**

Due to the risk of AmpC induction and subsequent emergence of resistance on therapy, IDSA guidelines suggest avoiding ceftriaxone (CRO) for the treatment of *E. cloacae, K. aerogenes* or *C. freundii* (***CEK*** group) but not for *S. marcescens (SM),* citing the lack of evidence. Many clinical laboratories still report CRO susceptibilities for these organisms. In January 2021, our institution stopped reporting CRO results for *Enterobacterales* with inducible AmpC enzymes. We compare rates of CRO treatment failure in the 2 years before and after this reporting change.

**Methods:**

We retrospectively evaluated (with IRB approval) all hospitalized patients that were diagnosed with an invasive infection due to CRO-susceptible *Serratia, Enterobacter, K. aerogenes,* or *C. freundii.* Final therapy was defined as antibiotic the patient was on within 24h of culture finalization for at least a 72h course. The primary outcome assessed rates of CRO treatment failure- defined as clinical failure (clinical decompensation) or microbiological failure (repeat growth of same organism). The years 2019 and 2020 (lab reported CRO) were compared to 2021 and 2022 (lab did not report CRO).

**Results:**

In 2019-2020, 67 patients were hospitalized with bacteremia, endocarditis, pneumonia or urosepsis (25 by *SM*, 42 by *CEK* group). 15 (22.4%) were treated with CRO (6 *SM,* 9 *CEK*). 12 patients (80%) failed CRO therapy (5 *SM,* 7 *CEK*), of which 4 had a repeat organism cultured, with 1 documented CRO-resistant isolate *(E. cloacae*) after treatment. In 2021-2022, of the 77 patients with infections (34 by *SM,* 43 by *CEK* group), 2 (2.6%) were treated with CRO and both failed therapy (2 *SM*).

**Table 1.**
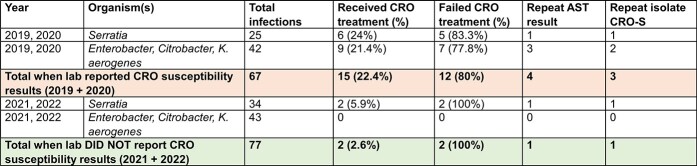
Summary of results. * CRO treatment failure rates based on number of patients who showed clinical or microbiology failure after being placed on CRO treatment

**Conclusion:**

Our single-center evaluation suggests that laboratories not reporting CRO susceptibility results for *Enterobacterales* with inducible AmpC may decrease use of CRO for these organisms and decrease subsequent treatment failures. Larger multi-center studies are warranted. Contrary to the IDSA guidelines, *SM* infections had a similar risk of CRO treatment failure as infections by *CEK* organisms, despite the initial isolate’s susceptibility. We hypothesize that due to the complex mechanism of inducible AmpC resistance, most cases of CRO treatment failure are not accompanied with the repeat culturing of a CRO-resistant organism after therapy.

**Disclosures:**

**Romney Humphries, PhD, D(ABMM), M(ASCP)**, Melinta: Advisor/Consultant|Merck: Advisor/Consultant|Shionogi: Advisor/Consultant|Ventorx: Advisor/Consultant

